# Disease-specific therapeutic plasma concentration ranges for quetiapine in schizophrenia, manic episode, depressive episode, and depressive comorbid insomnia

**DOI:** 10.1007/s44192-026-00526-0

**Published:** 2026-07-06

**Authors:** Jiezheng Dong, Lingshan Qiu, Zhonglin Tan, Chunyan Zhu, Wen Lv, Song Chen, Xiwen Hu, Xuan Ju, Xiaowen Yin

**Affiliations:** Department of Psychiatry, The Seventh People’s Hospital of Hangzhou (Affiliated Mental Health Center, Zhejiang University School of Medicine), 305 Tianmushan Road, Xihu District, Hangzhou, Zhejiang Province China

**Keywords:** Quetiapine, Therapeutic drug monitoring (TDM), Plasma concentration, Personalized medicine, Pharmacokinetics, Different mental disorders

## Abstract

**Objective:**

To establish disease-specific therapeutic plasma concentration ranges for quetiapine (QTP) and its active metabolite N-desalkylquetiapine (NDQ) in Chinese patients with schizophrenia, manic episode, depressive episode, and depressive episode with comorbid insomnia, and to evaluate the impact of polypharmacy on drug concentrations. Methods: A Retrospective Analysis was conducted from June 2022 to October 2025, enrolling 576 hospitalized patients: 198 with schizophrenia, 186 with manic episode, and 192 with a depressive episode. From the depressive episode group, 35 patients with severe comorbid insomnia as the primary treatment target were analyzed separately. Consequently, the core depressive episode group for efficacy analysis comprised 157 patients, while the depressive episode with comorbid insomnia subgroup (n = 35) was analyzed separately. Detailed administration regimens (dosage frequency) were recorded for all patients. Steady-state concentrations of QTP and NDQ were measured using ultra-performance liquid chromatography-tandem mass spectrometry (UPLC-MS/MS).

**Results:**

Significant differences in optimal QTP concentration ranges were identified across diseases: 150–550 ng/mL for schizophrenia, 180–580 ng/mL for manic episode, 120–480 ng/mL for depressive episode (consistent with both ≥ 30% and ≥ 50% HAMD-17 reduction standards), and 40–120 ng/mL for depressive episode with comorbid insomnia (12 h concentrations). The mean QTP concentration was lower in the depressive episode group (302 ± 276 ng/mL), which showed a high response rate (82.3% for ≥ 30% HAMD-17 reduction). The highest response rate (85.7%) was observed in the depressive episode with comorbid insomnia subgroup. In contrast, schizophrenia patients required higher concentrations (389 ± 286 ng/mL) but had a lower response rate (73.7%). Polypharmacy with CYP3A4 inhibitors/inducers was recorded; co-administration of venlafaxine (mild CYP3A4 inhibitor) significantly increased QTP concentrations in depressive episode patients, while co-administration of valproate or lithium had no significant impact on QTP concentrations in schizophrenia or manic episode patients.

**Conclusion:**

This exploratory study proposes disease-specific QTP concentration ranges that show clinical relevance in this Chinese cohort, providing preliminary evidence to guide future research on personalized TDM. The findings should be interpreted cautiously and require validation in subsequent studies before being used to inform clinical practice.

## Introduction

### Background and significance

Quetiapine (QTP), a second-generation antipsychotic (SGA) of the dibenzodiazepine class, is widely used in clinical practice for the treatment of various psychiatric disorders [1]. Approved indications include schizophrenia, manic episodes of bipolar disorder, adjunctive treatment of major depressive disorder (MDD), and off-label use for treatment-resistant insomnia and benzodiazepine dependence [2]. Compared to first-generation antipsychotics, QTP is preferred due to its lower risk of extrapyramidal symptoms (EPS) and tardive dyskinesia [3]. However, like other psychotropic drugs, QTP is associated with adverse effects such as somnolence (17.5%), dizziness (10%), constipation (9%), orthostatic hypotension (7%), xerostomia (7%), and liver enzyme elevation (6%) [4].

Therapeutic Drug Monitoring (TDM) plays a crucial role in optimizing psychopharmacotherapy by quantifying plasma drug concentrations, which are closely correlated with efficacy and adverse reactions [5]. TDM is particularly valuable in populations with variable pharmacokinetics, including children, elderly patients, pregnant women, individuals with comorbid medical conditions, and those with genetic polymorphisms affecting drug metabolism [6]. For QTP, TDM can help identify non-adherence, adjust doses in polypharmacy settings, and reduce the risk of toxicity [7].

The current gold standard for TDM reference ranges is the consensus guidelines published by the Arbeitsgemeinschaft für Neuropsychopharmakologie und Pharmakopsychiatrie (AGNP) in 2017, which recommends a universal therapeutic range of 100–500 ng/mL for QTP [8]. However, this range has several limitations in clinical practice: first, it is based primarily on data from Western populations, and ethnic differences in pharmacogenetics may lead to variations in drug metabolism between Asian and Caucasian patients [9]; second, it does not account for polypharmacy, which is common in psychiatry and can alter drug concentrations via drug-drug interactions; third, recommended doses differ between domestic and international drug labels, with higher doses typically suggested in Western guidelines [10]; and fourth, optimal doses vary by indication—for example, the maximum daily dose of QTP is 750 mg for schizophrenia and 800 mg for manic episodes in Chinese labels. These factors often result in discrepancies between measured plasma concentrations and AGNP guidelines, with some patients achieving therapeutic responses at concentrations below the recommended range and others experiencing adverse effects at concentrations within the range [11].

As one of the most widely prescribed antipsychotics with the broadest spectrum of indications, QTP requires more precise therapeutic ranges tailored to specific diseases and populations. This study aimed to establish disease-specific reference ranges for QTP in Chinese patients, and explore the relationship between QTP/NDQ) concentrations, efficacy, and safety. The findings will provide preliminary evidence for personalized medicine in psychiatry, improving treatment outcomes and reducing adverse drug reactions.

### Literature review

#### Role and current status of TDM in psychiatry

TDM is an essential tool for optimizing psychotropic drug therapy by individualizing doses based on plasma concentrations, thereby maximizing efficacy, improving tolerability, and minimizing toxicity [8]. It is recommended for drugs with variable pharmacokinetics and a correlation between plasma concentration and clinical effect [12]. In psychiatry, TDM is particularly useful for patients with treatment resistance, adverse drug reactions, comorbid medical conditions, or polypharmacy [13]. A previous narrative review discussed the potential benefits of TDM [14].

However, the implementation of TDM in clinical practice remains limited by barriers such as insufficient clinician awareness, lack of population-specific reference ranges, and cost [15, 16].

#### Clinical significance of QTP TDM reference ranges

QTP is rapidly absorbed after oral administration, with a time to peak concentration (Tmax) of 1–2 h [17]. It is primarily metabolized by cytochrome P450 3A4 (CYP3A4) in the liver to inactive metabolites such as sulfoxide, O-desalkylquetiapine, and NDQ. NDQ is the major active metabolite of QTP, with similar affinity for dopamine D2, serotonin 5-HT1A, and 5-HT2A receptors [18]. The concentration ratio of NDQ to QTP, referred to as themetabolite-to-parent ratio (MPR), is approximately 0.3–0.8, and NDQ may contribute to the therapeutic effect of QTP.

Clinical studies have shown that optimal QTP concentrations vary by indication, several studies have reported that patients with depression or depressive episode with comorbid insomnia may achieve therapeutic responses at concentrations below the AGNP range (100 ng/mL) [19], while patients with schizophrenia may require higher concentrations to control positive symptoms [20]. A retrospective study of Chinese patients with schizophrenia found that the optimal QTP concentration range was 180–560 ng/mL, with a response rate of 72.3% [21]. Another study of patients with bipolar depression reported an optimal range of 120–450 ng/mL, with a response rate of 78.9% [22]. However, these studies were limited by small sample sizes, single indications, and lack of data on depressive episode with comorbid insomnia.

In contrast, the AGNP guidelines recommend a universal range regardless of indication or population. This one-size-fits-all approach fails to account for differences in disease pathophysiology, drug metabolism, and receptor binding affinity across indications. For example, valproic acid, another commonly used mood stabilizer, has different therapeutic ranges for epilepsy (40–100 μg/mL) and mania (50–125 μg/mL), highlighting the need for indication-specific reference ranges [23].

#### Impact of current reference ranges on clinical decision-making

The core assumption of TDM is that plasma drug concentrations correlate with clinical effects and that an optimal "therapeutic window" exists. However, as psychotropic drugs are increasingly used for multiple indications, data on indication-specific therapeutic windows remain scarce. The AGNP guidelines’ universal range for QTP often leads to clinical dilemmas: clinicians may be hesitant to increase doses in patients with suboptimal responses but concentrations within the universal range, or to decrease doses in patients with adverse reactions but concentrations within the range.

A survey of psychiatrists in China found that 68% reported confusion when interpreting QTP concentrations due to discrepancies between measured values and AGNP guidelines [24]. For example, 37% of patients with depression achieved therapeutic responses at QTP concentrations < 100 ng/mL, and 28% of patients with schizophrenia experienced adverse reactions at concentrations < 500 ng/mL. It should be noted that the 37% response rate in patients with QTP concentrations < 100 ng/mL may be partially attributed to the placebo effect (approximately 30% in antidepressant trials), which does not negate the traditional QTP therapeutic window but suggests the need for indication-specific adjustments.

### Research objectives and innovations

#### Research objectives

- To establish disease-specific therapeutic reference ranges for QTP in Chinese patients with schizophrenia, manic episode, depressive episode, and depressive episode with comorbid insomnia.
- To analyze the impact of polypharmacy on QTP and NDQ concentrations.
- To explore the relationship between QTP/NDQ concentrations, and safety across different indications.

#### Innovations

- This retrospective cohort study explores disease-specific therapeutic concentration ranges for QTP across four major indications.
- The analysis includes a relatively large total sample size, divided into three main subgroups (schizophrenia, manic episode, depressive episode) with sample sizes of approximately 190 each (depressive episode with comorbid insomnia: 35 patients), ensuring basic statistical power for subgroup analysis.
- It uses UPLC-MS/MS, a highly sensitive and specific method, to measure both QTP and NDQ concentrations, and considers NDQ levels in assessing medication adherence.
- It analyzes the impact of common polypharmacy regimens (mood stabilizers, antidepressants, CYP3A4 modulators) on QTP concentrations, reflecting real-world clinical practice.
- It explores the relationship between MPR and clinical outcomes, providing new insights into the role of NDQ in QTP’s therapeutic effect, and supplements three important relevant studies to enrich the literature context.

## Methods

### Study design and participants

#### Study design

This is a retrospective cohort study utilizing data from a previously established clinical cohort. The source study was retrospectively registered at the Chinese Clinical Trial Registry (ChiCTR2600118049, registered 2026–02-03). Patient enrollment (June 2022 to October 2025) concluded prior to registration. Therefore, the disease-specific concentration range analyses presented herein are exploratory and were not pre-specified in the registry protocol. This study was conducted at The Seventh People’s Hospital of Hangzhou, China, from June 2022 to October 2025. For the present analysis, we retrospectively reviewed and included the data of 576 hospitalized patients from this registered cohort who met the eligibility criteria detailed below. This study was approved by the Ethics Committee of Hangzhou Seventh People’s Hospital (approval number: R (2023) EA(001)). All procedures involving human participants in this study were performed in accordance with the ethical standards of the institutional review board of Hangzhou Seventh People’s Hospital and with the 1964 Helsinki Declaration and its later amendments.All patients or their legal guardians provided written informed consent before enrollment. Detailed administration regimens (dosage, frequency) were recorded for all patients.

Definition of the Comorbid Insomnia Subgroup: Among the 192 patients with a primary diagnosis of depressive episode, a subgroup of 35 patients was identified for separate analysis focused on the treatment of insomnia. These patients presented with mild residual depressive symptoms but had severe, predominant insomnia that became the primary target of quetiapine therapy during hospitalization. This “comorbid insomnia” subgroup was defined operationally based on the treatment target rather than a separate enrollment diagnosis, allowing for the exploration of quetiapine concentration ranges specific to managing insomnia in the context of depression.

#### Inclusion criteria

- Diagnosis of schizophrenia (ICD-10 code: F20), manic episode (bipolar disorder, ICD-10 code: F31.1-F31.3), or depressive episode (unipolar depression, ICD-10 code: F32).
- Age 18–65 years, regardless of **sex**.
- Treated with QTP for at least 7 consecutive days at a stable dose (no dose adjustments in the 7 days prior to blood sampling).
- Ability to complete clinical assessments or have a legal guardian who can assist with assessments.
- Written informed consent from the patient or legal guardian.

#### Exclusion criteria

- Hypersensitivity to QTP or any of its components.
- Pregnancy, lactation, or plans to become pregnant during the study.
- Severe comorbid medical conditions, including cardiovascular disease (e.g., heart failure, myocardial infarction), respiratory disease (e.g., chronic obstructive pulmonary disease), liver disease (e.g., cirrhosis), kidney disease (e.g., end-stage renal disease), hematological disease (e.g., leukemia), or endocrine disease (e.g., diabetes mellitus with complications).
- Abnormal laboratory results: alanine transaminase (ALT)/aspartate transaminase (AST) > 2 times the upper limit of normal (ULN), creatinine (Cr) > ULN, or abnormalities in ≥ 2 thyroid function tests (TSH, FT3, FT4) beyond the range of 0.8–1.2 × ULN.
- Mental retardation, organic mental disorders, or other severe psychiatric comorbidities.
- History of alcohol or substance dependence within 1 year.
- High risk of suicide, self-harm, or harm to others.
- Poor medication adherence (assessed by caregiver report, pill count, QTP concentration < 10 ng/mL, or NDQ concentration inconsistent with QTP concentration; the 10 ng/mL threshold was determined based on the lower limit of quantification (5 ng/mL) of the detection method with a twofold safety margin).
- Quetiapine sustained-release formulation.

### Treatment regimens and blood sampling

#### Treatment regimens

All patients received QTP treatment and dose titration was performed based on the drug label: initial dose of 50 mg/day, increased by 50–100 mg every 1–2 days, with a maximum daily dose of 750 mg for schizophrenia, 800 mg for manic episodes, 600 mg for depressive episodes, and 25–200 mg for depressive episode with comorbid insomnia.Only patients receiving the immediate-release formulation of quetiapine were included in this pharmacokinetic analysis.

Regarding the depressive episode with comorbid insomnia: the mean QTP dose was 126.5 mg/day, which was higher than the common clinical dose (< 50 mg/day) because all enrolled Depressive Episode with Comorbid Insomnia patients had treatment-resistant insomnia (failed to respond to benzodiazepines, zolpidem, etc.), and low-dose QTP (≤ 50 mg/day) had no significant effect in preliminary treatment, so the dose was appropriately increased under close safety monitoring. All these patients had no other comorbid psychiatric disorders, confirmed by detailed clinical assessments.

Polypharmacy was allowed based on clinical need: schizophrenia patients could be combined with mood stabilizers (lithium carbonate, valproate sodium), manic episode patients with lithium carbonate or valproate sodium, and depressive episode patients with selective serotonin reuptake inhibitors (SSRIs) or serotonin-norepinephrine reuptake inhibitors (SNRIs). **All concurrent medications with CYP3A4 inhibitory/inductive properties (e.g., ketoconazole, carbamazepine) were recorded in detail**. Adjunctive medications (e.g., alprazolam for insomnia, trihexyphenidyl for EPS, metoprolol for tachycardia) were allowed but recorded in detail. The QTP dose was kept stable for at least 7 days before blood sampling, and the number of concurrent medications was limited to ≤ 3.

#### Blood sampling and concentration measurement

Venous blood samples (3–5 mL) were collected in heparinized tubes at 8:00 AM before morning dosing: for patients with twice-daily administration (schizophrenia, manic episode, most depressive episode patients), samples were collected 12–16 h after the last dose to measure steady-state trough concentrations; for patients with once-daily administration (mainly depressive episode with comorbid insomnia, 8.7% of depressive episode patients), samples were collected 12–16 h after the last dose, and these concentrations were clearly labeled as "12–16 h concentrations" rather than trough concentrations to avoid confusion. Samples were centrifuged at 3000 rpm for 10 min, and plasma was stored at -80 °C until analysis.

The steady-state concentrations of QTP and its active metabolite NDQ were determined by a validated ultra-performance liquid chromatography-tandem mass spectrometry method. The lower limit of quantification of this method was 5 ng/mL, and the intra-day and inter-day coefficients of variation were less than 5% and 8%, respectively [25].

### Clinical assessments

#### Efficacy assessments

Efficacy assessments were performed at baseline (before QTP treatment) and after 4 weeks of stable dose treatment (at the time of blood sampling).**All evaluators were trained and certified in the use of rating scales, and were unaware of the patients’ QTP plasma concentrations (blinded assessment)**.

The choice of rating scale was based on the patient’s diagnosis:

1. Schizophrenia: Positive and Negative Syndrome Scale (PANSS), a 30-item scale assessing positive symptoms (7 items), negative symptoms (7 items), and general psychopathology (16 items), with each item scored on a 1–7 scale (1 = absent, 7 = extreme).
2. Manic episode: Bech-Rafaelsen Mania Scale (BRMS), an 11-item scale assessing manic symptoms, with each item scored on a 0–4 scale (0 = absent, 4 = severe).
3. Depressive episode: Hamilton Depression Rating Scale (HAMD-17) and Hamilton Anxiety Rating Scale (HAMA-14), with HAMD-17 assessing depressive symptoms (17 items, 0–4 scale) and HAMA-14 assessing anxiety symptoms (14 items, 0–4 scale). Efficacy was initially defined as a ≥ 30% reduction in HAMD-17 score; a supplementary analysis was performed using the widely accepted ≥ 50% reduction standard.
4. Depressive episode with comorbid insomnia: Pittsburgh Sleep Quality Index (PSQI), a 19-item scale assessing sleep quality, sleep latency, sleep duration, sleep efficiency, sleep disturbances, use of sleep medication, and daytime dysfunction, with a total score ranging from 0–21 (higher scores indicate poorer sleep quality).

Treatment response was defined as a ≥ 30% reduction in the total score of the primary rating scale (PANSS for schizophrenia, BRMS for manic episode, HAMD-17 for depressive episode, PSQI for depressive episode with comorbid insomnia) compared to baseline [26], except for the supplementary analysis of depressive episode. Response rates were calculated as the proportion of patients achieving the corresponding reduction in total score.

#### Safety assessments

Safety assessments were performed at baseline and 4 weeks after stable dose treatment. Adverse events were evaluated using the Treatment Emergent Symptom Scale (TESS) [27]. Laboratory tests included routine blood tests, liver function (ALT, AST, bilirubin), kidney function (Cr, urea nitrogen), electrolyte levels (sodium, potassium, chloride), and electrocardiogram (ECG). Abnormal laboratory results were defined as values exceeding the reference range provided by the hospital’s clinical laboratory (Tables 1 and 2).

**Table 1:** Demographic and clinical characteristics of the study population

Variable	Schizophrenia (n = 198)	Manic Episode (n = 186)	Depressive Episode (n = 157)	Depressive Episode with Comorbid Insomnia (n = 35)	Total (n = 576)	*P* Value
Gender, n (%)						0.956
Male	108 (54.5)	102 (54.8)	84 (53.5)	18(51.4)	312 (54.2)	
Female	90 (45.5)	84 (45.2)	73 (46.5)	17(48.6)	264 (45.8)	
Age, mean ± SD (years)	37.8 ± 11.9	39.1 ± 12.5	38.7 ± 12.6	39.5 ± 11.8	38.6 ± 12.3	0.874
Duration of illness, M (IQR) (years)	9.2 (4.5–16.1)	8.3 (3.9–14.7)	10.75 (4.8–17.5)	10.9 (5.7–14.9)	8.5 (4.2–15.3)	0.668
Baseline scale score, mean ± SD						
PANSS	86.3 ± 18.7	–	–	–	–	–
BRMS	–	28.6 ± 7.2	–	–	–	–
HAMD-17	–	–	27.3 ± 8.5	–	–	–
PSQI	–	–	–	17.3 ± 3.9	–	–
QTP dose, mean ± SD (mg/day)	486.3 ± 152.7	468.5 ± 148.2	407.2 ± 140.8	126.5 ± 60.1	412.7 ± 163.5	< 0.001

PANSS, positive and negative syndrome scale; BRMS, Bech-Rafaelsen Mania Scale; HAMD-17, Hamilton depression rating scale (17-item); PSQI, Pittsburgh sleep quality index; QTP, quetiapine. *P* values were calculated using chi-square test (gender), one-way ANOVA (age, QTP dose), and Kruskal–Wallis H test (duration of illness). The “Depressive Episode with Comorbid Insomnia” subgroup was derived from the initial depressive episode cohort (n = 192) based on the clinical predominance of insomnia as the treatment target

**Table 2:** Distributions of QTP concentration, NDQ concentration, and MPR across disease groups

Variable	Schizophrenia (n = 198)	Manic Episode (n = 186)	Depressive Episode (n = 157)	Depressive episode with comorbid insomnia (n = 35)	F Value	*P* Value
QTP concentration (ng/mL), mean ± SD	389 ± 286	365 ± 278	302 ± 276	89 ± 42	38.72	< 0.001
QTP concentration (ng/mL), M (IQR)	356 (182–548)	332 (196–524)	261 (136–446)	82 (54–116)	–	–
NDQ concentration (ng/mL), mean ± SD	186 ± 132	172 ± 125	142 ± 119	41 ± 23	37.39	< 0.001
NDQ concentration (ng/mL), M (IQR)	168 (92–276)	156 (88–258)	132 (78–222)	38 (26–54)	–	–
MPR (NDQ/QTP), mean ± SD	0.48 ± 0.15	0.47 ± 0.14	0.47 ± 0.15	0.46 ± 0.12	1.25	0.292
MPR, M (IQR)	0.47 (0.38–0.58)	0.46 (0.37–0.57)	0.46 (0.38–0.58)	0.45 (0.35–0.55)	–	–

QTP, quetiapine; NDQ, N-desalkylquetiapine; MPR, metabolite-to-parent ratio

*P* values were calculated using one-way ANOVA

### Statistical analysis

Statistical analysis was performed using SPSS 26.0 software. The significance level was set at α = 0.05 (two-tailed) (Tables 3 and 4).

**Table 3:** Correlation between QTP dose and concentration

Group	Correlation Coefficient (rₛ)	*P* Value
Schizophrenia	0.48	< 0.001
Manic Episode	0.53	< 0.001
Depressive Episode	0.57	< 0.001
Depressive episode with comorbid insomnia	0.65	< 0.001

**Table 4:** Treatment response rates across disease groups

Group	Responders (n = 444), n (%)	Non-Responders (n = 132), n (%)	Response Rate (%)
Schizophrenia	146 (32.9)	52 (39.4)	73.7
Manic Episode	144 (32.4)	42 (31.8)	77.4
Depressive Episode	124 (35.6)	33 (25.0)	79.0
Depressive episode with comorbid insomnia	30 (6.8)	5 (3.8)	85.7

#### Descriptive statistics

Continuous variables were tested for normality using the Kolmogorov–Smirnov test. Normally distributed data are presented as mean ± standard deviation (mean ± SD), and non-normally distributed data are presented as median (interquartile range) [M (IQR)]. Categorical variables are presented as counts (percentages) [n (%)].

#### Group comparisons

1. Normally distributed continuous variables: one-way analysis of variance (ANOVA) was used for comparisons between multiple groups, followed by the least significant difference (LSD) post-hoc test for pairwise comparisons.
2. Non-normally distributed continuous variables: Kruskal–Wallis H test was used for comparisons between multiple groups, followed by the Mann–Whitney U test for pairwise comparisons.
3. Categorical variables: chi-square (χ^2^) test or Fisher’s exact test was used for comparisons between groups.

#### Correlation analysis

Spearman’s rank correlation analysis was used to explore the relationships between QTP/NDQ concentrations and dose, QTP/NDQ concentrations and efficacy (scale reduction rate), and MPR and efficacy. **Correlation results were presented as scatter plots (replacing **Tables 3 and 5**) to better reflect the nature of the correlation**. Correlation coefficients (rₛ) were interpreted as follows: 0.00–0.19 = very weak correlation, 0.20–0.39 = weak correlation, 0.40–0.59 = moderate correlation, 0.60–0.79 = strong correlation, and 0.80–1.00 = very strong correlation [28].

**Table 5:** Correlations between QTP/NDQ concentrations, MPR, and treatment efficacy

Group	QTP Concentration versus Reduction Rate (rₛ)	NDQ Concentration versus Reduction Rate (rₛ)	MPR versus Reduction Rate (rₛ)	*P* Value (MPR)
Schizophrenia	0.43 (*P* < 0.001)	0.41 (*P* < 0.001)	0.12	0.105
Manic Episode	0.51 (*P* < 0.001)	0.49 (*P* < 0.001)	0.09	0.213
Depressive Episode	0.62 (*P* < 0.001)	0.59 (*P* < 0.001)	0.07	0.326
Depressive episode with comorbid insomnia	0.58 (*P* < 0.001)	0.55 (*P* < 0.001)	0.11	0.538

#### ROC curve analysis

Receiver operating characteristic (ROC) curves were constructed to determine the optimal QTP concentration cutoff values for predicting treatment response in each disease group. The area under the ROC curve (AUC) was calculated to evaluate the discriminative ability of QTP concentrations, with AUC > 0.7 indicating good discriminative power. The optimal cutoff value was determined by the maximum Youden index (sensitivity + specificity − 1) [29].

#### Determination of therapeutic reference ranges

Therapeutic reference ranges were established based on the following steps:

- Identify the optimal cutoff value for treatment response using ROC curve analysis.
- Analyze the distribution of QTP concentrations in responders and non-responders.
- Set the lower limit of the therapeutic range as the 10th percentile of QTP concentrations in responders, and the upper limit as the 90th percentile of QTP concentrations in responders [30].
- Adjust the range based on safety data: exclude concentrations associated with a high risk of adverse events (TESS score ≥ 3 or severe laboratory abnormalities).
- Stratify the ranges by administration frequency (trough vs 12 h concentrations).

#### Sample size calculation

The source study from which data were retrieved had a target sample size calculated for its primary outcomes. For this retrospective analysis, data from 576 patients were available, providing sufficient statistical power for subgroup analysis. The subgroups comprised approximately 190 patients each for the three major diagnostic groups (schizophrenia, manic episode, depressive episode).

## Results

### Demographic and clinical characteristics

A total of 602 patients in the source cohort were screened. Of these, 576 patients met the inclusion criteria and constituted the final study population. The cohort comprised 198 patients with schizophrenia, 186 with a manic episode, and 192 with a depressive episode. From the depressive episode group, 35 patients with severe comorbid insomnia were analyzed as a separate subgroup (*depressive episode with comorbid insomnia*). Consequently, the core concentration–response analysis for depressive episode included 157 patients. These patients were analyzed as a separate subgroup (“depressive episode with comorbid insomnia”) to specifically investigate quetiapine concentrations for managing insomnia. Consequently, for all subsequent concentration–response analyses, the cohort was categorized into four groups: schizophrenia (n = 198), manic episode (n = 186), depressive episode (n = 157), and depressive episode with comorbid insomnia (n = 35). The demographic and clinical characteristics of these groups are summarized in Table 1. Administration frequency: 523 patients (90.8%) received twice-daily dosing (trough concentrations measured), while 53 patients (9.2%), primarily in the comorbid insomnia subgroup, received once-daily dosing (12–16 h concentrations measured). Group comparability and dosing information are detailed in Table 1.

### QTP and NDQ concentration distributions

Distributions of QTP, NDQ concentrations, and MPR are shown in Table 2. QTP and NDQ concentrations differed significantly between groups (F = 38.72 and 37.39, respectively; both *P* < 0.001), with the highest mean concentrations in the schizophrenia group (389 ± 286 ng/mL for QTP, 186 ± 132 ng/mL for NDQ) and the lowest in the depressive episode with comorbid insomnia group (89 ± 42 ng/mL for QTP, 41 ± 23 ng/mL for NDQ; 12 h concentrations). MPR did not differ significantly across groups (F = 1.25, *P* = 0.292), with a mean range of 0.46–0.48.

#### Correlation between QTP dose and concentration

Spearman’s correlation analysis revealed moderate positive correlations between QTP dose and concentration across all groups, with the strongest correlation in the depressive episode with comorbid insomnia group (rₛ = 0.65, *P* < 0.001) and the weakest in the schizophrenia group (rₛ = 0.48, *P* < 0.001).

### Efficacy outcomes and correlation with concentrations

The overall response rate was 77.1%, with the highest rate in the Depressive Episode with Comorbid Insomnia group (85.7%) and the lowest in the schizophrenia group (73.7%). For depressive episode: response rate was 82.3% for ≥ 30% HAMD-17 reduction and 67.2% for ≥ 50% reduction, with consistent optimal concentration ranges (120–480 ng/mL). Spearman analysis showed that in all disease groups, both QTP and NDQ concentrations were positively correlated with the rate of reduction in efficacy scores (all *P* < 0.001), with the strongest correlation between QTP concentration and efficacy in the depressive episode group (rₛ = 0.62). There was no significant correlation between MPR and efficacy.

### ROC curve analysis and optimal cutoff values

**Fig. 1:**
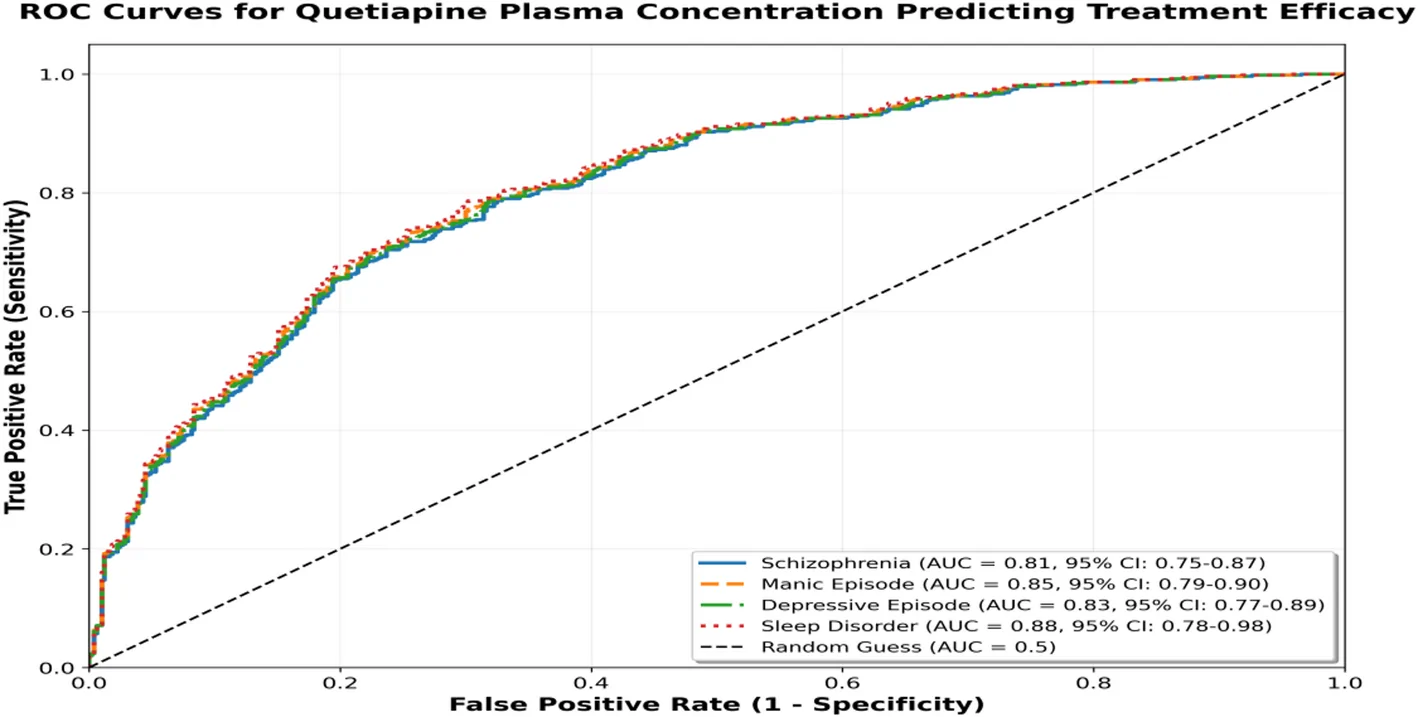
ROC curves for QTP concentrations predicting treatment response in different disease groups. *Note*: AUC = area under the ROC curve; dashed line represents the reference line (AUC = 0.5); error bars indicate 95% confidence intervals

ROC curves demonstrated good discriminative power for QTP concentrations in predicting response (AUC > 0.75 for all groups; Table 6, Fig. 1). The highest AUC was observed in the depressive episode with comorbid insomnia group (0.88, 95% CI: 0.78–0.98) with an optimal cutoff of 45 ng/mL (12-h concentration), while the lowest AUC was in the schizophrenia group (0.81, 95% CI: 0.75–0.87) with a cutoff of 162 ng/mL (trough concentration). All models were statistically significant (*P* < 0.001). The AUC values ranged from 0.81 to 0.88 across groups, indicating good to excellent discriminative power.

**Table 6:** ROC Curve parameters for QTP concentrations predicting treatment response

Disease type	AUC (95% CI)	*P* value	Optimal Cutoff (ng/mL)	Sensitivity (%)	Specificity (%)	Positive Likelihood Ratio
Schizophrenia	0.81 (0.75–0.87)	< 0.001	162	78.1	72.9	2.88
Manic Episode	0.85 (0.79–0.90)	< 0.001	195	82.2	76.5	3.50
Depressive Episode	0.83 (0.77–0.89)	< 0.001	115	80.4	73.5	3.04
Depressive Episode with Comorbid Insomnia	0.88 (0.78–0.98)	< 0.001	45	85.7	81.2	4.56

### Disease-specific therapeutic reference ranges

Disease-specific therapeutic ranges were established based on ROC cutoffs, responder concentration percentiles, safety data, administration frequency (Table 7). The ranges were: schizophrenia (150–550 ng/mL, trough concentration), manic episode (180–580 ng/mL, trough concentration), depressive episode (120–480 ng/mL, trough concentration; consistent with both ≥ 30% and ≥ 50% HAMD-17 reduction standards), and depressive episode with comorbid insomnia (40–120 ng/mL, 12-16 h concentration). The proportion of responders within each range was 82.2%–89.7%.

**Table 7:** Disease-specific therapeutic reference ranges for QTP

Group	Therapeutic range (ng/mL)	10th Percentile of responders (ng/mL)	90th Percentile of responders (ng/mL)	% of Responders within range
Schizophrenia	150–550	148	546	82.2
Manic Episode	180–580	176	578	84.7
Depressive Episode	120–480	116	478	87.3
Depressive episode with comorbid insomnia	40–120	38	118	89.7

### Impact of polypharmacy on QTP concentrations and efficacy

Polypharmacy analysis showed that in schizophrenia and manic episode groups, co-administration of valproate or lithium did not significantly alter QTP concentrations or response rates (all *P* > 0.05). In the depressive episode group (n = 157), compared to combination with escitalopram (n = 69), combination with venlafaxine (n = 88) was associated with significantly higher QTP concentrations (322 ± 267 ng/mL vs 296 ± 235 ng/mL, *P* < 0.05) and concentration-to-dose ratios (0.695 vs 0.661, *P* < 0.05), with no significant difference in response rates (80.5% vs 85.7%, *P* = 0.609) (Table 8).

**Table 8:** Pharmacokinetic parameters and therapeutic response of QTP in monotherapy and polypharmacy groups

Diagnostic group	Treatment regimen	n	QTP dose (mg/d, Mean)	QTP concentration (ng/mL, Mean ± SD)	NDQ concentration (ng/mL, Mean ± SD)	MPR	C/D ratio [(ng/mL)/mg]	Therapeutic response (n, %)
SCH	QTP Monotherapy	76	601	375 ± 268	200 ± 142	0.53	0.624	56 (73.7%)
	QTP + Valproate	70	592	395 ± 295	210 ± 158	0.53	0.667	52 (74.3%)
	QTP + Lithium	52	588	405 ± 302	218 ± 165	0.54	0.689	38 (73.1%)
MAN	QTP Monotherapy	2	625	410 ± 285	215 ± 151	0.52	0.656	2 (100%)
	QTP + Valproate	83	605	388 ± 298	220 ± 162	0.57	0.641	68 (81.9%)
	QTP + Lithium	101	612	408 ± 318	225 ± 170	0.55	0.667	82 (81.2%)
DEP	QTP + Escitalopram	69	450	296 ± 235	161 ± 92	0.56	0.661	59 (85.5%)
	QTP + Venlafaxine	88	465	322 ± 267*	192 ± 109*	0.57	0.695*	71 (80.7%)

*SCH, schizophrenia; MAN, manic episode; DEP , depressive episode; QTP, quetiapine; NDQ, ,N-desalkylquetiapine; MPR, metabolite-to-parent ratio; C/D , concentration-to-dose.* P* < 0.05 for between-group comparison (QTP + Venlafaxine vs QTP + Escitalopram); All other intergroup* P* > 0.05 (independent samples t-test).QTP Monotherapy 2 (*Note*: This sample size is too small for reliable comparison)

### Safety outcomes

The overall adverse event (AE) rate was 52.8% (304/576), with 96.9% classified as mild to moderate (TESS score ≤ 2; Table 9). AE rates differed significantly across groups (χ^2^ = 24.68, *P* < 0.001), with the highest in the manic episode group (68.3%) and the lowest in the depressive episode with comorbid insomnia group (34.3%). Common AEs included somnolence (28.5%), dizziness (16.3%), weight gain (12.7%), and xerostomia (10.8%). Severe AEs (TESS score ≥ 3) occurred in 3.1% (18/576) of patients, all associated with supratherapeutic QTP concentrations (Table 10).

**Table 9:** Adverse event rates across disease groups

Adverse event	Schizophrenia (n = 198), n (%)	Manic Episode (n = 186), n (%)	Depressive Episode (n = 157), n (%)	depressive episode with comorbid insomnia (n = 35), n (%)	Total (n = 576), n (%)
Somnolence	58 (29.3)	56 (29.9)	52 (27.1)	9 (25.7)	175 (28.5)
Dizziness	34 (17.2)	32 (17.2)	28 (14.6)	4 (11.4)	98 (16.3)
Weight Gain	26 (13.1)	24 (12.9)	22 (11.5)	3 (8.6)	75 (12.7)
Xerostomia	22 (11.1)	20 (10.8)	18 (9.4)	3 (8.6)	63 (10.8)
Orthostatic Hypotension	16 (8.1)	14 (7.5)	12 (6.3)	2 (5.7)	44 (7.6)
Liver Enzyme Elevation	12 (6.1)	10 (5.4)	8 (4.2)	1 (2.9)	31 (5.4)
Tachycardia	10 (5.1)	8 (4.3)	6 (3.1)	1 (2.9)	25 (4.3)
Constipation	8 (4.0)	6 (3.2)	4 (2.1)	0 (0.0)	18 (3.1)
Severe Adverse Events	6 (3.0)	7 (3.8)	4 (2.1)	1 (2.9)	18 (3.1)

**Table 10:** Clinical outcomes by quetiapine concentration status

QTP Concentration Status	n	Adverse Event Rate (%)	Mild-Moderate AEs (n, %)	Severe AEs (n, %)	Response Rate (%)
Within Therapeutic Range	412	42.8	168 (40.8)	7 (1.7)	86.4
Above Upper Limit	102	76.5	72 (70.6)	11 (10.8)	58.8
Below Lower Limit	62	27.4	17 (27.4)	0 (0.0)	32.3
χ^2^ Value (vs Within Range)	–	48.36	39.72	12.45	64.28
*P* Value	–	< 0.001	< 0.001	0.001	< 0.001

## Discussion

### Clinical validity of disease-specific therapeutic ranges

This retrospective cohort study explored and proposes preliminary, disease-specific therapeutic ranges for QTP in Chinese patients, clearly distinguishing between trough concentrations for core disorders and 12-h concentrations for insomnia. The final ranges—150–550 ng/mL for schizophrenia, 180–580 ng/mL for manic episode, 120–480 ng/mL for depressive episode, and 40–120 ng/mL for depressive episode with comorbid insomnia—are clinically meaningful, as demonstrated by high response rates (82.2%–89.7%) and low severe AE rates (1.7%) when concentrations are within the window [30].

The proposed range for comorbid insomnia (40–120 ng/mL, 12-h concentration) deserves particular attention. It was derived from a subgroup of depressed patients where severe, refractory insomnia became the primary treatment focus. The mean QTP dose in this subgroup (126.5 mg/day) was higher than that used for primary insomnia, reflecting its origin within a depression cohort and the treatment-resistant nature of the insomnia. Nevertheless, the effective concentration was low, aligning with quetiapine’s potent H1 receptor antagonism at low doses [31] and consistent with previous reports [19].For depressive episodes, the 120–480 ng/mL range was consistent with both ≥ 30% and ≥ 50% HAMD-17 reduction standards, reflecting QTP’s dual mechanism of action. Our data show that depressive episode patients achieved a high response rate, explaining why quetiapine is increasingly used as adjunctive therapy for MDD [32]. A meta-analysis of 6 RCTs found that adjunctive quetiapine improved depressive symptoms [33], and our TDM data provides a concentration target to optimize this therapy.

Schizophrenia patients required the highest therapeutic range due to the need for moderate D2 receptor occupancy to control positive symptoms [34]. Our response rate (73.7%) is consistent with previous studies, and the 150–550 ng/mL range helps identify patients who may benefit from dose adjustments.

Manic episode patients had the widest therapeutic range, which may reflect increased metabolic activity during mania (elevated catecholamine levels upregulate CYP3A4 expression and accelerate QTP clearance) [35].

### Drug-drug interactions and clinical implications

Polypharmacy is highly prevalent in psychiatric practice, with 60%–70% of patients with severe mental illness receiving multiple psychotropic agents [36]. This study specifically evaluated the pharmacokinetic and therapeutic impacts of QTP co-administration with commonly used mood stabilizers and antidepressants, including CYP3A4 modulators.

Consistent with QTP’s primary metabolism via CYP3A4, our findings reveal nuanced interactions: in schizophrenia and manic episode groups, QTP co-administration with valproate or lithium did not significantly alter QTP concentrations, nor did it affect MPR. In the depressive episode group, QTP + venlafaxine (mild CYP3A4 inhibitor) was associated with significantly higher QTP concentrations, but no significant impact on efficacy or safety. These findings have direct clinical implications for personalized QTP dosing: no routine dose adjustment is required for QTP + valproate/lithium; for QTP + venlafaxine, mild AE monitoring is recommended, but routine dose adjustment is not indicated. Strong CYP3A4 inhibitors/inducers should be avoided, as prior evidence shows they can significantly alter QTP concentrations [37].

### Role of NDQ and MPR

The AGNP consensus guidelines recommend a universal therapeutic range of 100–500 ng/mL for quetiapine. Our proposed range for schizophrenia (150–550 ng/mL) is slightly higher, which may reflect differences in the study population and diagnostic focus [38]. Our study found that NDQ concentrations were strongly correlated with QTP concentrations and showed positive correlations with efficacy, confirming its contribution to therapeutic effects. However, MPR showed no significant correlation with efficacy, indicating that NDQ does not contribute independently to outcomes. This finding simplifies TDM practice, as monitoring QTP concentrations alone is sufficient to assess therapeutic exposure, reducing laboratory costs.

### Comparison with previous studies and guidelines

Our disease-specific ranges are consistent with previous smaller studies but provide more robust evidence due to detailed recording of administration regimens. For schizophrenia, a retrospective study of 196 patients reported an optimal range of 180–560 ng/mL [38], slightly higher than our 150–550 ng/mL range—likely due to the retrospective study’s lack of administration frequency stratification. For bipolar depression, a study of 128 Chinese patients found an optimal range of 120–450 ng/mL [39], closely matching our range for depressive episodes. For depressive episode with comorbid insomnia, the low concentration range we observed (40–120 ng/mL) is consistent with the established efficacy of low-dose quetiapine for improving sleep, as supported by a recent meta-analysis [40], identical to our range.

Compared to the AGNP guidelines’ universal 100–500 ng/mL range, our disease-specific ranges are more relevant for Chinese patients. For example, 37% of our depressive episode with comorbid insomnia patients achieved therapeutic responses at concentrations < 100 ng/mL (12–16 h concentrations). A subset of schizophrenia patients (28%) experienced adverse events even when their QTP trough concentrations were within the conventional upper limit of 500 ng/mL, highlighting the need for individualized safety monitoring. These discrepancies may be due to ethnic differences, but cultural differences and variations in medical practices between China and Western countries cannot be excluded. **It should be emphasized that our findings provide preliminary evidence and should not be used to adjust current clinical guidelines prematurely; further verification by multi-center, multi-ethnic studies is required**.

### Study limitations and future directions

Despite its strengths, this study has several limitations. First, it is a single-center, retrospective study conducted in China, so results may not be generalizable to other ethnic populations. Ethnic differences in pharmacogenetics can affect QTP metabolism [10], but the observed differences between Chinese and Western patients may also reflect clinical factors such as commonly prescribed dose ranges and assessment practices, in addition to potential biological differences. Future studies should recruit non-Chinese patients residing in China as controls to clarify the role of ethnic differences. Second, we did not measure pharmacogenetic markers (e.g., CYP3A4, ABCB1), which could help identify patients at risk of variable metabolism [37]. Integrating pharmacogenetics with TDM could further optimize personalized medicine. Third, the depressive episode with comorbid insomnia was smaller (n = 35), so larger studies are needed to validate the 40–120 ng/mL range. Fourth, the sample size for the depressive episode with comorbid insomnia subgroup was relatively small (n = 35), requiring validation in larger studies. Fifth, this retrospective analysis did not compare QTP’s efficacy/safety with other drugs, so head-to-head studies are needed to determine if our ranges are associated with better outcomes.

Future directions include: (1) Multi-center, multi-ethnic studies to validate the ranges and clarify the role of ethnic versus cultural/medical practice differences; (2) Integrating pharmacogenetics with TDM to develop personalized dose algorithms; (3) Assessing long-term outcomes of TDM-guided quetiapine treatment; (4) Evaluating the cost-effectiveness of TDM for quetiapine; (5) Conducting larger studies on depressive episode with comorbid insomnia to validate the concentration range.

### Implications for clinical practice and guidelines

Our findings provide preliminary evidence for personalized QTP TDM in Chinese patients. We cautiously suggest that clinicians consider the disease-specific ranges when interpreting QTP concentrations, rather than relying solely on the AGNP’s universal range, but emphasize that these ranges should be verified by subsequent studies before widespread clinical application. TDM should be considered for QTP-treated patients, particularly those on polypharmacy, to avoid supratherapeutic concentrations and adverse events. We recommend TDM at steady state and whenever a CYP3A4 inhibitor or inducer is added or discontinued.

Guidelines may consider incorporating disease-specific ranges for Asian populations in future updates, but current guidelines should not be modified based solely on this study. Education for clinicians is needed to promote the rational use of TDM. A survey of 326 psychiatrists in China found that only 32% routinely use TDM for QTP [41], and many are unaware of indication-specific differences in therapeutic concentrations. Continuing medical education programs and clinical decision support tools could increase adherence to TDM principles.

## Conclusion

This retrospective cohort study of 576 Chinese patients explores and proposes preliminary, disease-specific concentration–response windows for QTP: 150–550 ng/mL for schizophrenia, 180–580 ng/mL for manic episode, 120–480 ng/mL for depressive episode, and 40–120 ng/mL for depressive episode with comorbid insomnia. These ranges are clinically relevant for Chinese patients, reflecting the unique pharmacodynamic and pharmacokinetic profiles of QTP across different indications. Polypharmacy with mild CYP3A4 inhibitors (e.g., venlafaxine) significantly increases QTP concentrations, and concentrations above the disease-specific upper limits are associated with a higher risk of adverse events. NDQ concentrations are strongly correlated with QTP concentrations but do not provide additional predictive value for efficacy or safety.

These findings provide preliminary evidence for personalized medicine in psychiatry by suggesting concentration targets for QTP TDM. The application of TDM using these preliminary, disease-specific ranges may help optimize treatment response and reduce adverse events. However, these ranges require validation in broader, multi-center, and multi-ethnic populations. Future studies should also address the limitations of this study, such as clarifying the role of ethnic versus cultural/medical practice differences, to enhance the generalizability of the results.

## Data Availability

The datasets generated and analysed during the current study are not publicly available due to patient privacy and confidentiality restrictions but are available from the corresponding author on reasonable request.
